# Perfusion and Diffusion Abnormalities of Multiple Sclerosis Lesions and Relevance of Classified Lesions to Disease Status

**DOI:** 10.4172/2155-9562.S12-012

**Published:** 2014-04-28

**Authors:** Lian Li, Michael Chopp, Siamak P. Nejad-Davarani, Kourosh Jafari-Khouzani, Suresh C. Patel, John Budaj, Mei Lu, Stanton B. Elias, Mirela Cerghet, Quan Jiang

**Affiliations:** 1 Department of Neurology, Henry Ford Hospital, Detroit, Michigan, USA; 2 Department of Physics, Oakland University, Rochester, Michigan, USA; 3 Department of Radiology, Henry Ford Hospital, Detroit, Michigan, USA; 4 Department of Biostatistics and Research Epidemiology, Henry Ford Hospital, Detroit, Michigan, USA

**Keywords:** Cerebral blood volume, Fractional anisotropy, Multiple sclerosis, Lesion load, Brain atrophy, Clinical disability, MRI

## Abstract

**Objective:**

Hemodynamic abnormality and disruption of white matter (WM) integrity are significant components in the pathophysiology of multiple sclerosis (MS) lesions. However, the roles of stratified lesions with distinct degrees of hemodynamic and structural injury in disease states remain to be explored. We tested the hypothesis that hemodynamic and structural impairment, as assessed by cerebral blood volume (CBV) and fractional anisotropy (FA), respectively, characterizes the extent of tissue injury, and the load of lesion with substantial tissue destruction would reflect the disease status and therefore, would be related to clinical disability.

**Methods:**

Seven relapsing-remitting MS patients and seven healthy controls underwent perfusion, diffusion and conventional MRI scans. Based on T2-FLAIR and T1-weighted image, WM plaques were classified. After image coregistration, values of CBV and FA were estimated in three distinct lesion types (active, T1-hypointense and T1-isointense lesion) and compared with those obtained in WM from controls. A total of 1135 lesions were evaluated. Brain volumetric measurement and correlative analysis between brain atrophy, lesion volume and clinical disability were also performed.

**Results:**

Compared with normal WM, significantly reduced CBV and FA were present in the T1-hypointense lesion, while insignificant changes in both parameters were exhibited in the T1-isointense lesion. However, increased CBV but significantly decreased FA was detected in the active lesion. A close spatial relationship between active and T1-hypointense lesion was observed. Lesion load represented by T1-hypointense plus active lesion volume significantly correlated with brain atrophy, which, in turn, significantly correlated with the severity of clinical disability.

**Conclusion:**

A distinct combination of CBV and FA characterizes the status of a specific lesion type. A severe structural impairment does not solely occur in the T1-hypointense lesion, but is also associated with the active lesion. The burden of the lesion with extensive structural damage provides an image index, indicative of disease status.

## Introduction

As a disease of the central nervous system, multiple sclerosis (MS) is the leading cause of non-traumatic neurological disability in young adults, affecting more than two million people worldwide [1]. Although the pathogenesis of the disease is not yet fully understood, hemodynamic abnormality [2,3] and disruption of white matter (WM) integrity [1,4,5] are associated with the pathological processes and contribute to the appearance of MS lesions.

Accumulating data show that MS discrete lesions do not instantly emerge, but gradually develop [2,6–9], and eventually lead to a wide variety of neurological disabilities due to irreversible axonal damage [1,4]. Classification of the lesions by the severity of tissue injury may provide crucial information for shaping therapeutic goals since the lesion at the chronic stage with extensive axonal loss [10] may be less responsive to current treatments than the lesion at the early stage with inflammation, edema and demyelination of axons [11]. Moreover, identification of lesions with severe tissue destruction could potentially serve as a surrogate marker for characterizing the disease status, which is accompanied with the corresponding clinical disability.

As an advanced imaging technique, magnetic resonance imaging (MRI) plays an important role in diagnosis and monitoring of the disease by non-invasively visualizing the focal lesions and revealing their hemodynamic and microstructural alterations in vivo [12]. However, with perfusion and diffusion measurements, most of previous image-based investigations have focused on either hemodynamic [3,13,14] or structural [15–17] abnormalities of MS lesions. Few studies [2,18] have analyzed specific lesions and examined both features, which are important components of the pathophysiology of MS lesions [9,19] and seem to be coupled temporally and spatially with the disease evolution [2]. Therefore, revealing the extent of tissue damage resulting from these destructive and interactive events [2,14,20–22] in all categories of lesions may provide insight into the roles of classified lesions in the disease state and the substrate of irreversible progressive disability in MS. Furthermore, increasing evidence suggests that volumetric MRI assessment of brain atrophy correlates with both physical disability and cognitive impairment in patients with MS [23,24], and lesion load has an impact on such a correlation [25]. Nevertheless, to our knowledge, the effects of different lesion types with distinct degrees of hemodynamic and structural injury on brain morphologic change remain to be systematically explored. Such investigation may help to elucidate the pathological processes responsible for disease progression. In addition, quantification based on image data derived from an echo-planar-imaging (EPI) sequence, such as dynamic susceptibility contrast (DSC) perfusion imaging and diffusion tensor imaging (DTI), is a methodological challenge due to the image distortion [26], particularly for MS lesions, which are scattered throughout the brain, with the majority of them being small in size. Manual processing of lesion data, like movement or adjustment of region-of-interest (ROI) on the basis of visual criteria, would bias the assessment results. Thus, there is a considerable interest in objectively and accurately depicting the MS lesions with specific damage status evaluated by both their hemodynamic and structural impairments. Meanwhile, the clinical relevance of these stratified lesions to disease progression, as indicated by brain atrophy and clinical dysfunction, needs to be established.

The purpose of this study was three-fold: 1) to semi-automatically identify and classify MS lesions using FLAIR image in combination with the T1-weighted image (T1WI), 2) to reveal their tissue injury features by quantifying the perfusion and diffusion abnormalities after image distortion correction, and 3) to test the hypothesis that hemodynamic and structural impairments, as assessed by cerebral blood volume (CBV) and fractional anisotropy (FA), respectively, characterize the extent of tissue injury, and the load of lesion with substantial tissue destruction would reflect the disease status and therefore, would be related to clinical disability.

## Materials and Methods

### Subjects

The patients were enrolled from MS Clinic at Henry Ford Hospital, and their Expanded Disability Status Scale (EDSS) scores [27] were evaluated by an MS specialist prior to the MRI study. A total of 25 patients participated in this study and underwent MRI scans. To reveal the perfusion and diffusion abnormalities among the different lesions, patients with all types of distinct MS lesions (i.e., presenting with contrast-enhanced lesions) were included. For this purpose, we studied seven patients with clinically definite relapsing-remitting MS [28] (all female; mean age 36.6 years, range 20–50 years; median duration of the disease 10.9 years, range 7–17 years; median EDSS score 4.0 points, range 1.0–8.0 points). Seven age- and gender-matched healthy volunteers (mean age 40.7 years, range 21–48 years) with no history of neurological disorder, brain injury, cerebrovascular disease or any marked intracranial pathology at MR imaging served as controls. Approval from the Henry Ford Health System Institutional Review Board and written informed consent from all the subjects were obtained prior to the study.

### MRI data acquisition

All imaging was performed using a clinical MR system (GE HD 3.0T, Healthcare, Waukesha, WI, USA) with an 8-channel brain array coil. The MRI protocol consisted of: 1) Pre- and post-contrast high-resolutionT1-weighted 3D IRSPGR imaging (TE/TI=3.5/500 ms, flip angle=150, FOV=240×240 mm, acquisition matrix=256×192, slice thickness=1.0 mm); 2) T2-FLAIR imaging (fluid attenuated inversion recovery, TR/TE/TI=9000/120/2250 ms, acquisition matrix=384×256); 3) DSC perfusion imaging (multiple gradient-echo single-shot EPI, TR/TE=1900/40 ms, flip angle=900, acquisition matrix=128×128, intravenous bolus injection of the Gd-DTPA (Magnevist: Bayer HealthCare Pharmaceuticals Inc., Wayne, NJ, USA): 25 mL at a rate of 4 mL/second, immediately followed by 20 mL of saline flush at the same rate); and 4) Diffusion imaging (multiple spin-echo single-shot echo-planer diffusion tensor sequence, TR/TE=8000/93 ms, diffusion weighting of b=1500 s/mm^2^ at 55 non-collinear gradient directions, additional acquisition of 6 T2-weighted volumes with non-diffusion weighting (b=0 s/mm^2^).

For T2-FLAIR, perfusion and diffusion scans, images were acquired in the axial plane using the same FOV of 240×240 mm, slice position and slice thickness of 4 mm with no interslice gap, contiguously covering the whole brain (T2-FLAIR and diffusion) or supratentorial brain (perfusion).

### Image processing and analysis

#### Maps generated from perfusion and diffusion measurements

Maps of CBV, a hemodynamic perfusion parameter, and FA, a tensor-derived diffusion index, were calculated with the methods that Rempp et al. [29] and Basserand Pierpaoli [30] described, respectively.

#### Lesion identification, classification and ROI creation

**Figure 1:**
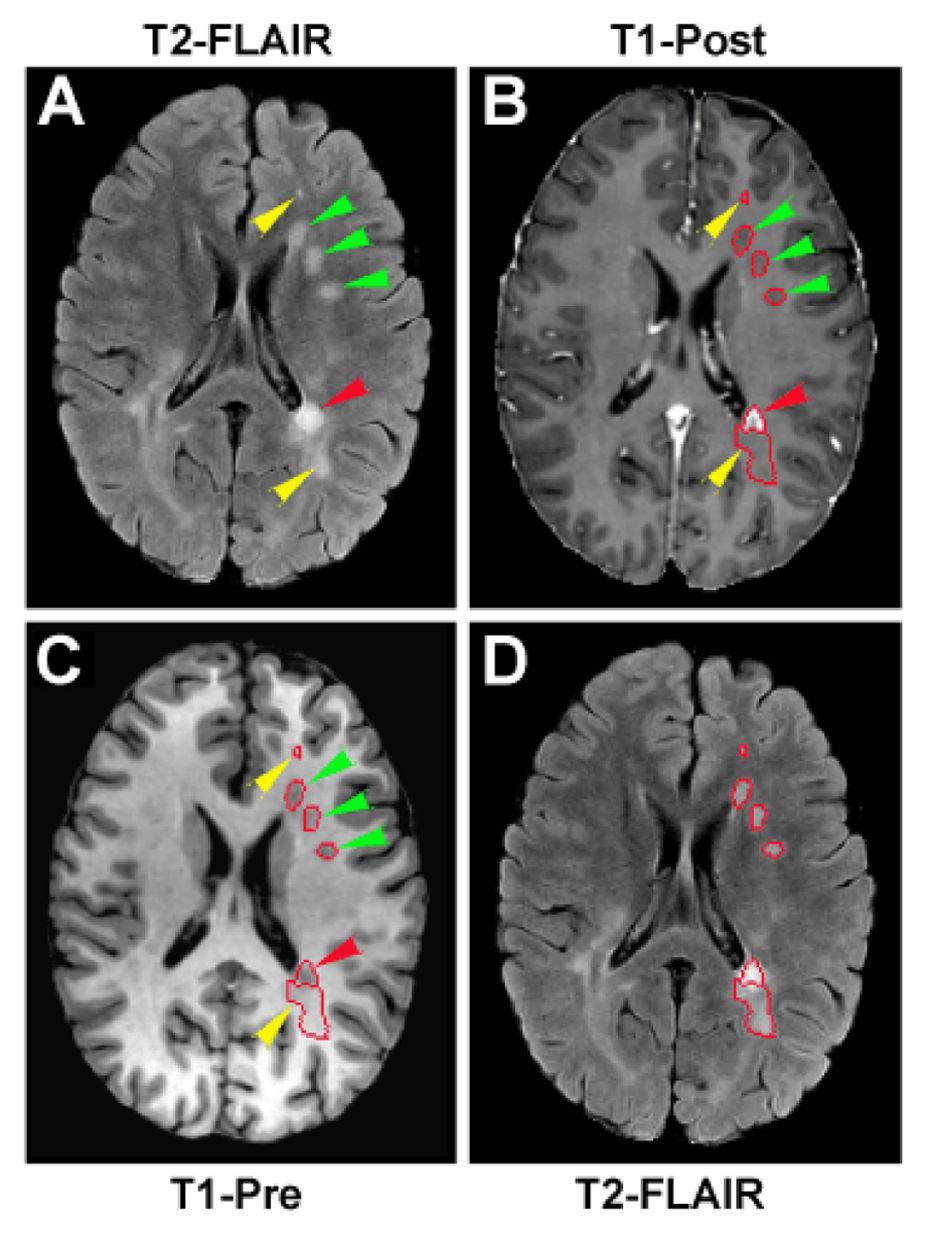
An axial section of T2-FLAIR (A and D) and T1WI (B and C) showing lesion classification and ROI creation. Abnormal hyperintensities detected on T2-FLAIR image (arrowheads in A) appear as enhanced (red arrowhead in B), hypointense (green arrowheads in B and C) or isointense (yellow arrowheads in B and C) areas compared to the surrounding NAWM on T1WI. T1-hypointense lesion (green arrowheads in C) was identified by the overlapped region of hyperintense area on T2-FLAIRimage (green arrowheads in A) and hypointense area on T1-Pre image (green arrowheads in C). While enhanced region on T1-Post image delineated the active lesion (red arrowhead in B), the overlapped region of hyperintense area on T2-FLAIR image (yellow arrowheads in A) and isointense area on T1-Pre image (yellow arrowheads in C) represented the T1-isointense lesion. The whole set of ROIs was created on T2-FLAIR image (red tracks on D) and applied to parametric quantification (To illustrate, ROIs of this axial section only in the left side of the brain were presented here).

**Figure 2:**
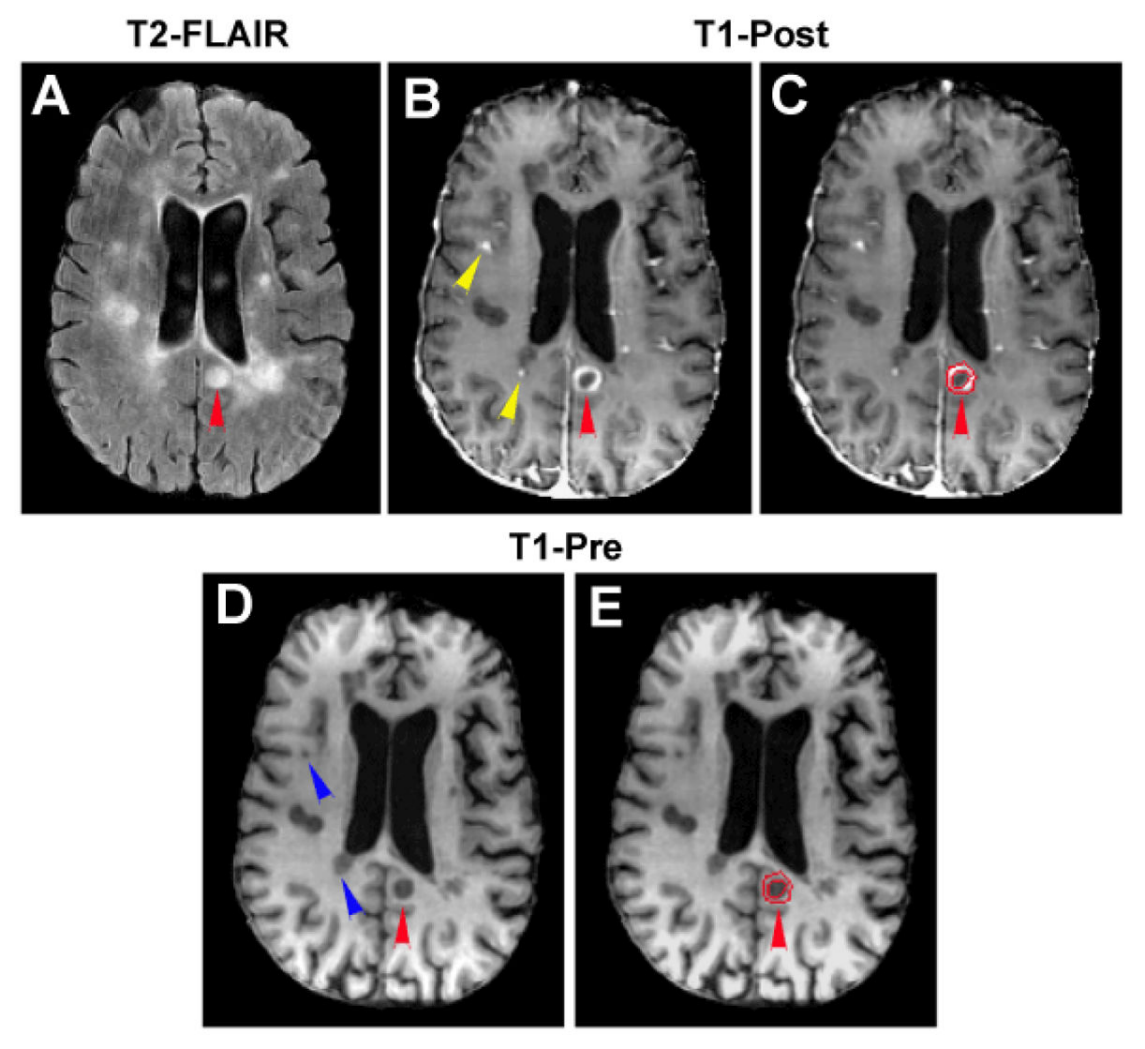
An axial section of T2-FLAIR (A), T1-Post (B and C) and T1-Pre (D and E) image showing a typical ring-enhanced lesion, its corresponding ROIs and the spatial relationship between active and T1-hypointense lesion. A hyperintense spot observed on T2-FLAIR image (red arrowhead in A) may represent a ring-enhanced lesion on T1-Post image (red arrowheads in B and C). It should be noted that remarkable T1 signal loss occurred inside the enhanced ring (E). The entire lesion area was then divided into two regions, T1-enhanced (C, the area encompassed by two red tracks) and T1-hypointense (C, the rest central part encompassed by the smaller red track) regions. Just like the ring-enhanced region surrounding the hypointense area, some active lesions appeared immediately adjacent to the T1-hypointense lesions (comparing the locations of enhanced regions indicated by yellow arrowheads in B and the locations of hypointense areas indicated by blue arrowheads in D).

With mutual information-based image registration (Eigentool image analysis software, Henry Ford Health System, MI, USA), T1WI was aligned onto T2-FLAIR image. Both T2-FLAIR image and T1WI were used to identify and classify MS lesions which caused a variety of signal changes on these images. As illustrated in Figure 1, abnormal hyperintensities detected on T2-FLAIR image (arrowheads in Figure 1A) could appear as enhanced (red arrowhead in Figure 1B), hypointense (green arrowheads in Figure 1B and 1C) or isointense (yellow arrowheads in Figure 1B and 1C) areas with respect to the surrounding normal appearing white matter (NAWM) on T1WI. The hyperintensities identified on the T2-FLAIR image were similar on the pre- and post-contrast T1WI, except the plaque area with Gd-DTPA enhancement (comparing Figure 1B with 1C). We, therefore, used the post-contrast T1WI (T1-Post) to depict the T1-enhanced or active lesion and the pre-contrast T1WI (T1-Pre) combined with T2-FLAIR image to classify the remaining lesions without enhancement. The hyperintensities on T2-FLAIR image were specified by those pixels with pixel value higher than the mean plus twice the standard deviation (mean+2SD) provided by the surrounding NAWM. Similarly, the hypointensities on T1-Pre image comprised those pixels with pixel value lower than the mean minus twice the standard deviation (mean-2SD) measured from the surrounding NAWM. The isointensities on T1-Pre image then indicated those pixels with less signal loss than the hypointensities defined by the local threshold. T1-hypointense lesion was identified by the overlapped region of hyperintense area on the T2-FLAIR image and hypointense area on the T1-Pre image. While the enhanced region on the T1-Post image delineated the active lesion, the overlapped region of hyperintense area on T2-FLAIR image and isointense area on T1-Pre image represented the T1-isointense lesion. For a ring-enhanced lesion (Figure 2), the bright ring area on T1-Post image, as defined above, depicted the T1-enhanced region, and the central part was identified as the T1-hypointense or T1-isointense region depending on its T1 value compared to the nearby NAWM on T1-Pre image.

The whole set of ROIs was created on T2-FLAIR image (Figure 1D) for each measured axial section and was applied to parametric quantification afterwards. The supratentorial lesions, representing the vast majority and common location of MS lesions in the brain [15,31], were analyzed in the present study.

The volume for a specific type of lesion in the whole supratentorial brain was obtained by adding all the areas of this lesion detected on individual sections and multiplying the total by the section thickness. Lesions with the area less than 5 pixels (~ 4.4 mm^2^) or in close proximity to cerebrospinal fluid (CSF) were excluded to eliminate partial volume artifact.

### Brain segmentation, volumetric measurement and WM ROIs

Based on acquired high resolution 3D images, the whole brain was segmented using HAMMER software (Section of Biomedical Image Analysis, University of Pennsylvania, PA, USA) and the masks representing the regions of WM, gray matter and ventricles (lateral, third and fourth ventricle) were obtained. The volumes of these regions were then calculated on the basis of their areas on individual sections and section thickness.

The axial sections of 3D images that matched the locations of its T2-FLAIR image were selected. For healthy controls, the WM masks on these selected sections were used as WM ROIs for quantifying CBV or FA, while for patients, these masks were copied onto their corresponding T2-FLAIR images to ensure that only those lesions located within the WM region were included in this study.

### Image distortion correction and parameter evaluation

To accurately perform quantification in the specific lesions (patients) and WM region (controls), the perfusion and diffusion image distortion induced by eddy currents has to be corrected. Despite the general promising results, mutual information-based matching still has its own limitations [32] which could be the reason for failure to rectify the distortion of our data. We therefore used an image registration software known as bUnwarpJ [33], an ImageJ plug in which was designed to align pairs of images distorted by both physical and acquisition related distortions, for the task. This approach combines the ideas of elastic and consistent image registration and minimizes the similarity error between the target and source image after imposing a consistency constraint. As a bidirectional registration, the algorithm calculates deformation fields for both direct (from source image to target image) and inverse (from target image to source image) transformation simultaneously.

**Figure 3:**
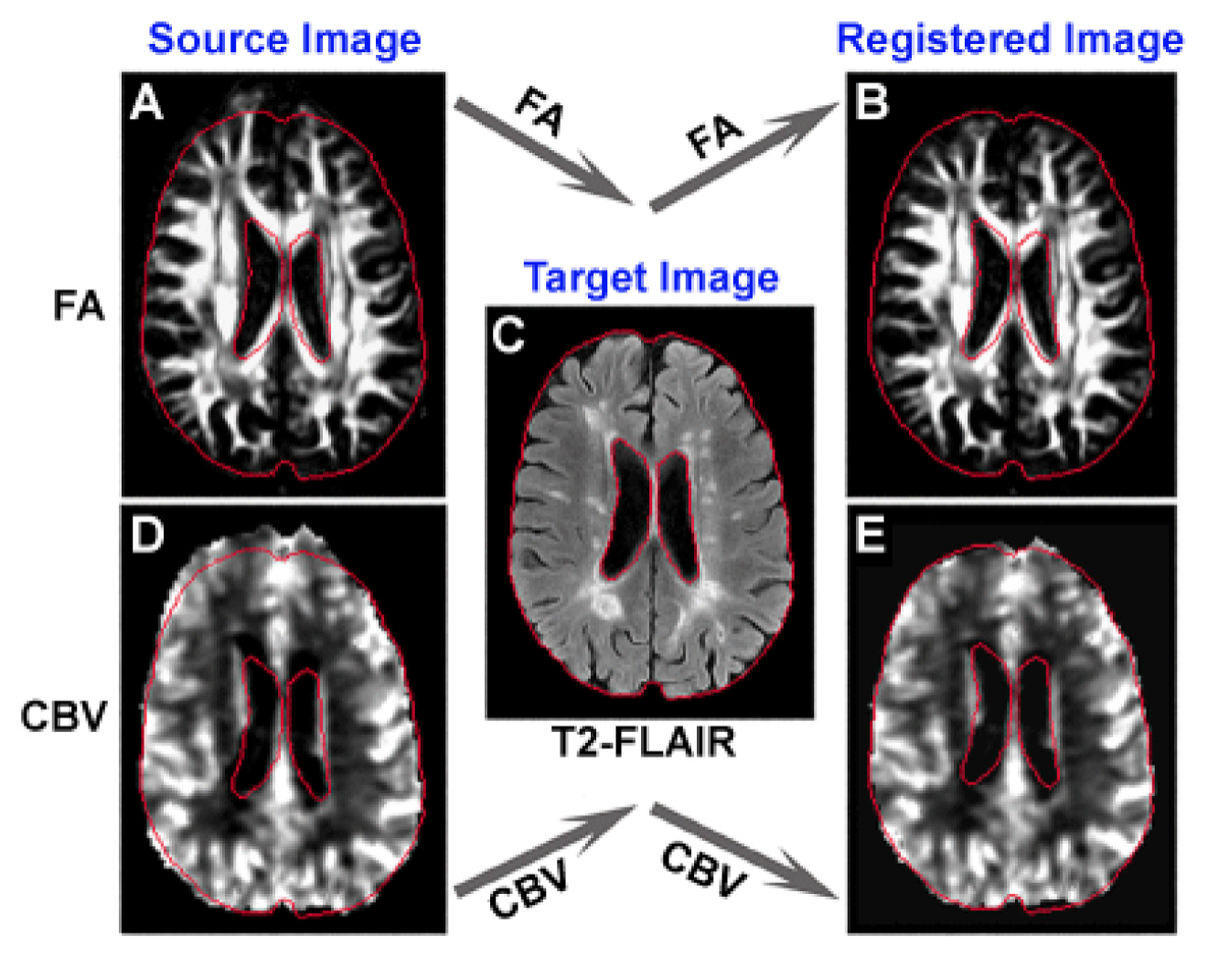
An axial section of FA (A and B), T2-FLAIR (C) and CBV (D and E) showing the registration results. A set of red tracks are the outlines of the brain and ventricles obtained from the T2-FLAIR image (C), which served as the target image during the registration. The mismatch between the outlines and the original FA (A) or CBV (D) illustrated a severe distortion, whereas a nice match between the outlines and the transformed FA (B) or CBV (E) demonstrated that the distortion was well corrected after the registration.

In practice, perfusion-weighted (pre-contrast injection) or diffusion-weighted (b=0) images were used as the source images, while T2-FLAIR or 3D images were the targeted images. The obtained deformation fields were applied for direct transformation of CBV or FA, respectively. The representative registration results are shown in Figure 3. Each section of original CBV or FA for all patients was aligned onto its corresponding target T2-FLAIR image on which lesion ROIs were created. Similarly, all sections of the original CBV or FA for all controls were registered onto their corresponding target 3D images, where WM region was delineated after segmentation.

Since the values of CBV and FA may change after registration procedure, the parameter evaluation was not performed on the registered image. Instead, the lesion and WM ROIs based on target image (T2-FLAIR or 3D image) were inversely transformed to source image (original CBV or FA) where the estimation was conducted for all sections studied.

A total of 1135 lesion spots were evaluated for MS patients. Among them, 50, 468 and 617 were identified as active, T1-hypointense and T1-isointense lesion, respectively. The WM regions on all supratentorial sections were assessed for healthy controls.

### Statistical analysis

Statistical analysis was performed using SAS (Cary, NC, USA, version 9.2). Data were evaluated for normality first, and a data transformation or nonparametric approach was considered if they were not normally distributed. Two-sample t-test was employed to detect the differences in MRI measurements (CBV, FA and brain volumetric results) between the different lesions and WM region and between the MS patients and healthy controls. The marginal regression was used to deal with multiple-slice evaluations per subject. Correlative analysis was conducted between lesion volume, ventricle size and clinical assessment in MS patients. The Pearson correlation coefficient was computed to provide a measure of the degree of linear association between the estimated factors. Results are presented as mean ± standard error (SE). Statistical significance was inferred for p ≤ 0.05.

## Results

### Spatial relationship between T1-enhanced and T1-hypointense region

Our observation revealed that the location of enhanced regions on T1-Post image was closely related to the site of hypointense areas on T1-Pre image. For 50 enhanced regions detected, the vast majority of them were either immediately adjacent to/surrounding (56%) or exactly at/overlapping (40%) the hypointense areas. Only 4% of them occurred at the T1-isointense regions on the T1-Pre image, and they appeared independent of hypointense areas.

**Figure 4:**
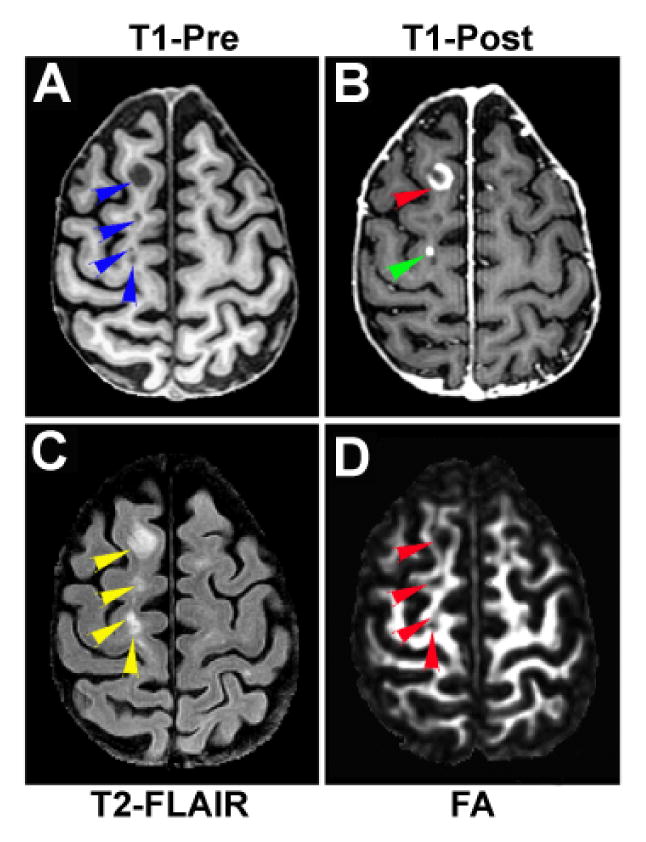
Relationship between T1-enhanced and T1-hypointense region. Some areas withT1-hypointense signal on T1-Pre image (arrowheads in A) concurrently, may be the T1-enhanced regions on T1-Post image (arrowheads in B). Both T1-enhanced and T1-hypointense regions present as hyperintensities on T2-FLAIR image (comparing A, B with C). The reduced FA regions (arrowheads in D) correspond not only to the T1-hypointense lesions, but also to the adjacent active (T1-enhanced) lesions (comparing A, B with D).

Figures 2 and 4 are representative illustrations of the relationship between the enhanced regions and the hypointense areas. The enhanced regions on T1-Post image (indicated by yellow arrowheads in Figure 2B) were located immediately next to the hypointense areas on T1-Pre image (indicated by blue arrowheads in Figure 2D), while a ring-enhanced region appeared directly encompassing a hypointense area (comparing Figure 2C with Figure 2E in the lesion area, as indicated by red arrowhead). Meanwhile, some areas with T1-hypointense signals on the T1-Pre image (arrows in Figure 4A) were also T1-enhanced regions on T1-Post image (arrows in Figure 4B). These observations illustrate that the active and T1-hypointense lesions are spatially related. Moreover, our imaging data showed that the reduced FA regions (arrows in Figure 4D), indicative of loss of the intensity or directionality of white matter fiber tracts, corresponded not only to the T1-hypointense lesions as expected, but also to the active lesions (comparing Figure 4A, 4B with 4D).

### CBV and FA in different types of lesions

**Figure 5:**
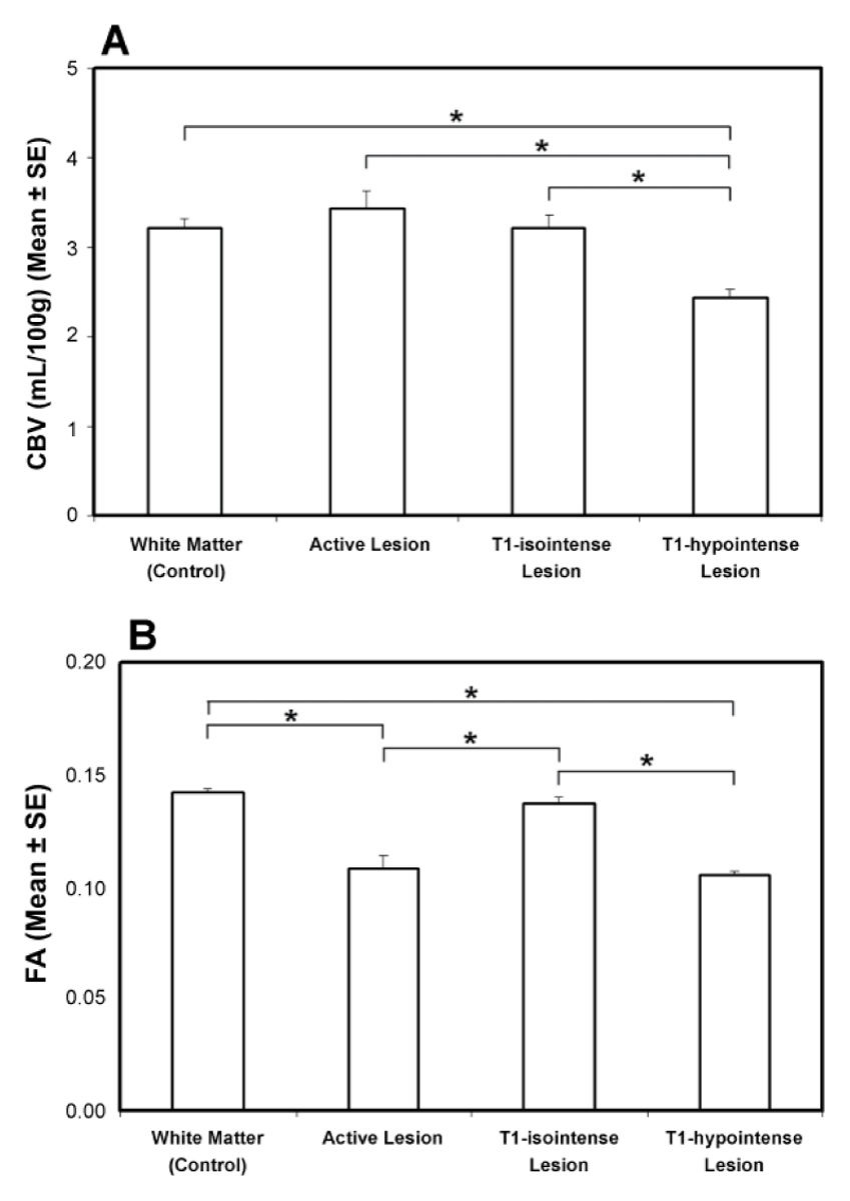
Quantitative results of CBV (A) and FA (B) in the WM of healthy controls and in the specific lesions of MS patients. Significantly lower CBV was found in the T1-hypointense lesion than in the rest of the lesion types and normal WM. Significantly lower FA was detected in both the T1-hypointense and active lesions than in the T1-isointense lesion and normal WM. *: p< 0.05.

In the four measured regions, significantly lower CBV (p<0.05) was found in the T1-hypointense lesion compared to the other regions (Figure 5A). No significant differences in CBV were detected among the active lesion, T1-isointense lesion and WM region of controls. Significantly reduced FA values (p<0.05) were detected in both T1-hypointense and active lesions compared to T1-isointense lesions and WM region of controls, while no significant differences in FA were found between T1-isointense lesions and WM region of controls and between the T1-hypointense and active lesions (Figure 5B).

### Brain volumetric change in patients with MS

**Figure 6:**
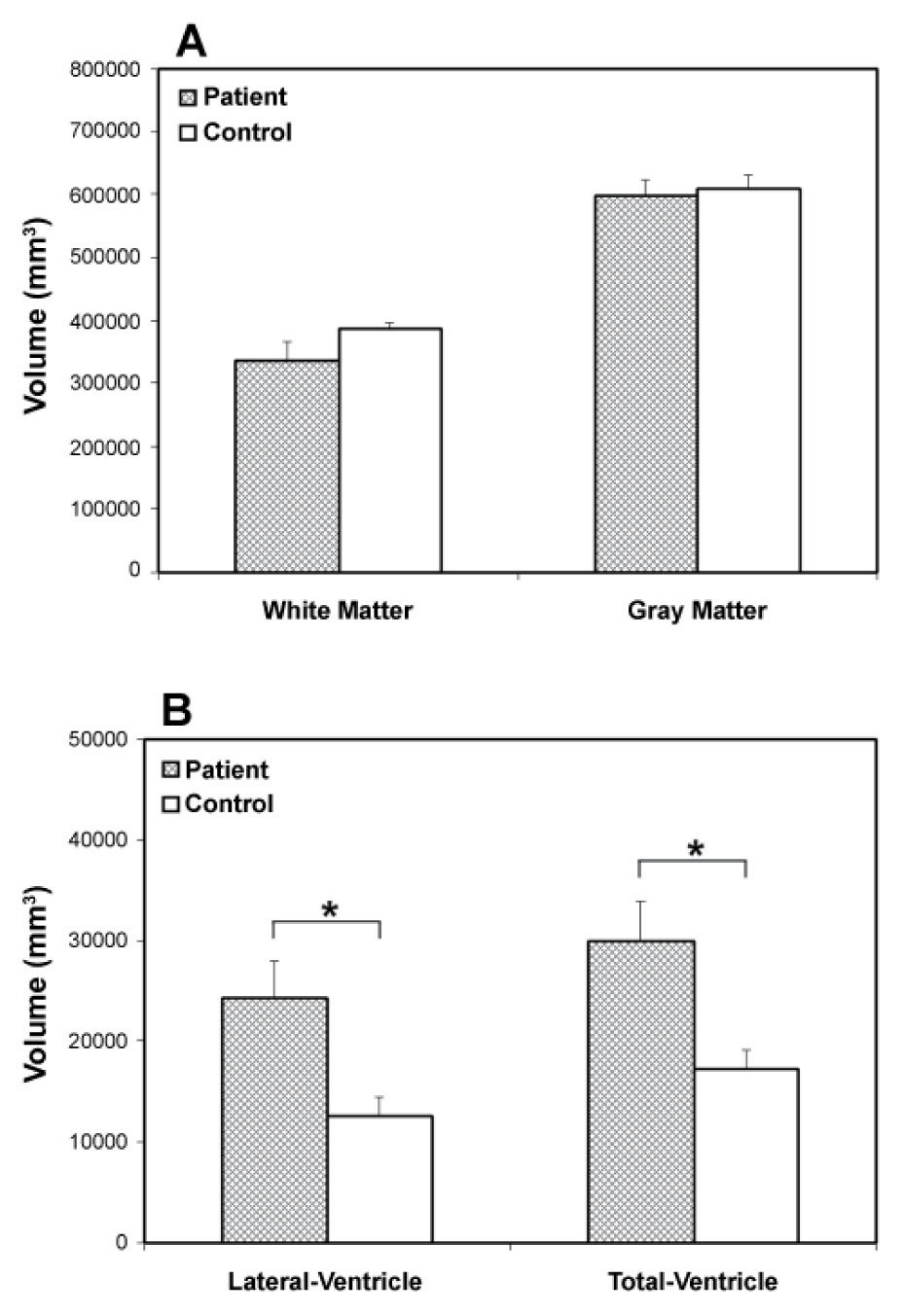
Volumetric data showing WM, gray matter and ventricle volume in the patient and control group. Lower WM and gray matter volumes in the patients than in the healthy controls were detected, although no significant differences between two groups were present (A). Significantly enlarged lateral and total ventricular volumes were found in the patient group compared to the control group (B).*: p< 0.05.

Volumetric data shown in Figure 6 revealed lower mean values of WM and gray matter volumes in the patients than in the healthy controls, although no significant differences between two groups were detected (Figure 6A). However, significantly expanded ventricles (p<0.05), such as lateral ventricle (right lateral ventricle +left lateral ventricle) and total ventricle (lateral ventricle + third ventricle + fourth ventricle), were found in the patient group compared to the control group (Figure 6B).

### Correlative analysis

**Figure 7:**
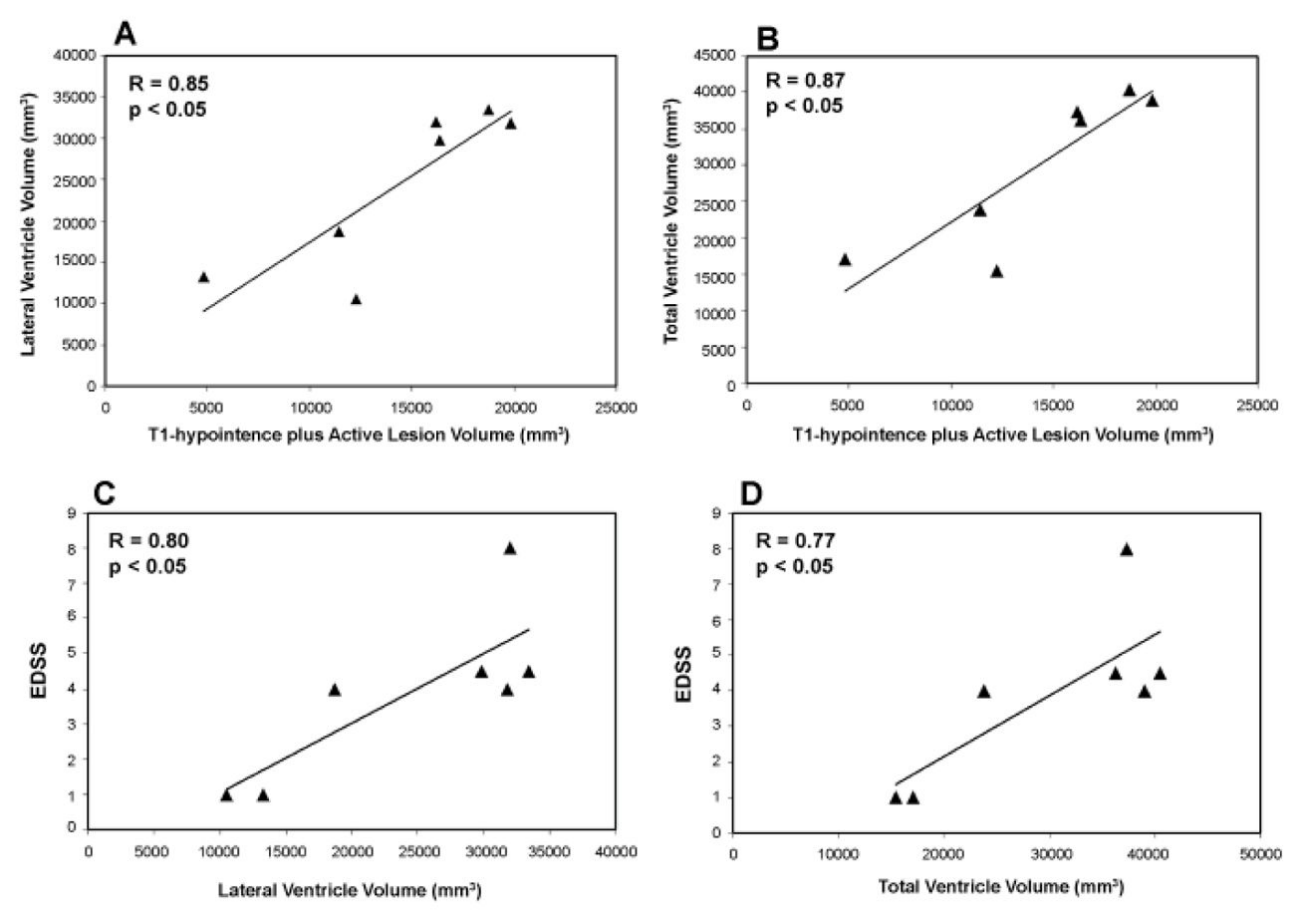
Scatter plots (with linear regression lines) showing the correlation between lesion volume and brain volumetric change (A, B), and between ventricular expansion or brain atrophy and disability status (C, D) in patients with MS.

To investigate the effect of lesion volume on brain volumetric change and further, the effect of brain atrophy on clinical disability status in patients with MS, correlative analysis was performed. As shown in Figure 7, T1-hypointense plus active lesion volume significantly correlated with lateral (Figure 7A; R=0.85, p<0.05) and total ventricle (Figure 7B; R=0.87, p<0.05) volume, with a larger lesion volume leading to a larger ventricular expansion. Lateral (Figure 7C; R=0.80, p<0.05) and total ventricle volume (Figure 7D; R=0.77, p<0.05) significantly correlated with EDSS, with a larger ventricle size or an increased brain atrophy resulting in a higher EDSS.

## Discussion

In the present study, we investigated hemodynamic and structural abnormalities in three categories of distinct MS lesions. We also explored the clinical relevance of these stratified lesions to disease state by revealing the relationship between lesion volume, brain atrophy and disability status. Our data showed that each specific lesion type presented a distinct combination of perfusion and diffusion alteration, as estimated by CBV and FA, respectively. A severe structural impairment did not solely occur in the T1-hypointense lesion, but was also associated with the active lesion. Importantly, lesion load represented by T1-hypointense plus active lesion volume significantly correlated with brain atrophy, which, in turn, significantly correlated with the severity of clinical disability, suggesting that a burden of the lesion with extensive tissue destruction appears to provide an image index, indicative of disease status.

While T2WI shows its remarkable sensitivity in detecting MS lesions, T1WI provides additional information on the same lesions regarding the pathological substrate, particularly for active and T1-hypointense lesions with ongoing blood-brain barrier (BBB) breakdown and substantial tissue destruction, respectively [10,34,35]. For supratentorial lesions, FLAIR image depicts them better than does T2WI [36]. We, therefore, used the T2-FLAIR image to identify the lesions, and combined it with T1WI when classifying the lesions into three specific types: active (T1-enhanced), T1-hypointense and T1-isointense lesion. To reduce human intervention, local threshold was employed throughout the procedure, and the lesion ROIs that encompassed the area with significant signal changes were created. For an accurate estimation, each section of CBV and FA was coregistered onto its corresponding conventional MRI, on which lesion or WM ROIs were produced.

In comparison with normal WM, T1-hypointense lesions presented with a significantly reduced CBV and FA, indicating severe hemodynamic and structural impairment. With the least chance to revert, T1-hypointense lesion, then, may be mainly responsible for permanent disability. There were no significant changes in CBV and FA in the T1-isointense lesion with respect to normal WM, whereas significant differences in both parameters between the T1-isointense and T1-hypointense lesion were detected, consistent with previous findings [14,16,37]. Hence, the T1-isointense lesion represents the pathology-affected but not seriously disturbed tissue with ongoing injury and self-repair, such as demyelination and remyelination [38]. Compared with normal WM, a significantly decreased FA was also found in the active lesion with elevated CBV, as expected [2]. These data might suggest an effect of compensatory vasodilatation accompanying inflammatory activity [3,13,14], and indicate that a similar structural disruption occurs in both T1-hypointense and active lesions. These data are interpreted and supported by other groups’ estimates [15,17] and our observation showing a close spatial relationship between T1-hypointense and active lesions. Results of a prior study [35] demonstrate that the majority of the active lesions (80%) on T1-Post image exhibit hypointensities on T1-Pre image, corresponding to the areas with axonal damage or dysfunction, as evidenced by significantly reduced concentration of N-acetyl derived metabolites [34]. In addition, it has been noted that axonal injury extends beyond the MS plaque borders seen on the conventional MRI, occurring to a greater extent in the areas adjacent to plaques than in WM regions remote from plaques [39,40]. Thus, it is likely that an active lesion appears in the place where a considerable microstructural impairment is present. Advanced tissue destruction is not limited to the T1-hypointense lesion areas but broadens into their periphery regions. In agreement with these reports, our data showed that a high percentage of the active lesions (96%) were found at (40%) or adjacent to (56%) the T1-hypointense lesions, where structural disorganization occurred as reflected by diminished FA. Although some factors related to the active lesion, such as accumulation of inflammatory cells and myelin breakdown products, could potentially restrict water diffusion and decrease FA due to the presence of nonoriented barriers to diffusion, the effect is likely small because of their relatively low concentrations [16].

There is a large body of evidence demonstrating that destructive pathological processes, such as hypoperfusion, demyelination and axonal damage, are far from limited to discrete lesions, but also occur in the NAWM in patients with MS [41–44]. The widespread loss of tissue integrity in the NAWM leads to a global cerebral atrophy, which is associated with the disease progression [23–25]. Our data confirm the fact that ventricular expansion or brain atrophy is correlated with the worsening clinical disability in MS. More importantly, our measurements also revealed a significant correlation between T1-hypointense plus active lesion volume (a burden of the lesion with severe tissue destruction) and brain atrophy, implying that irreversible tissue damage is an important determinant of disease progression. There are complex pathogenetic mechanisms leading to the alteration of NAWM in patients with MS. Of the mechanisms that could cause the loss of tissue integrity in the NAWM, Wallerian degeneration of axons transected by remote, but connected focal lesions may play an important role [43]. MRI and postmortem investigations demonstrate that the extent of axonal injury in specific areas of the corpus callosum (NAWM) is associated with the lesion load in the cerebral lobes most connected to those regions [45,46]. Thus, the local lesion has a remote impact on the tissue damage in NAWM via Wallerian degeneration. The heavier lesion load appears in the brain, the higher risk of diffuse axonal injury would occur, which accounts for the subsequent cerebral atrophy and clinical disability. However, less information exists regarding the effects of lesion types characterized by their distinct hemodynamic and structural impairment on morphologic brain changes. The present study revealed that not the total lesion load, but a joint burden of MS lesion (T1-hypointense plus active lesion volume) significantly correlated with ventricular enlargement or global atrophy of the brain, indicating that the load of lesion with substantial tissue disruption has a determinant impact on diffuse brain injury. These data are consistent with the idea that “black holes” and active inflammation are the significant factors in the pathogenesis of whole brain atrophy in MS [47–50]. Except for brain atrophy, however, any type or combination of lesion load was not found to directly correlate with clinical status. These results support the previous finding that brain atrophy is a clinically relevant measure in MS [24], and meanwhile, suggest that the load of lesion with extensive tissue destruction provides an assessment of the disease burden.

T1 enhancement is considered as the marker of active inflammation with BBB disruption in MS and therefore, is used to assess disease activity [51]. The number and volume of enhanced or active lesions are strong short-term predictors of subsequent disability [52]. The appearance of active lesions in patients with secondary progressive MS has been found to link to more rapid FA decline in the corpus callosum during 1-year observation period than in patients without active lesions [53]. Other investigations, though not all [54], also reach the conclusion that a larger number of T1 enhancement corresponds to a greater degree of disease progression [55–57]. These findings support an association between progressive brain damage and inflammatory disease activity [53]. In agreement with this hypothesis, our data indicate that the active lesion does play a role in disease status, and contributes to brain atrophy and probably to disease progression.

We are aware of limitations of the present study. The numbers of patients with three categories of lesions presented on MRI scan enrolled in the study were small. This can be offset, at least partially, by a large number of lesions evaluated. As mentioned above, we estimated the supratentorial lesions slice by slice consecutively, which included 50 measurements for the active lesion alone and a large number of measurements for the T1-hypointense (468) and T1-isointense (617) lesion. Secondly, we used the entire supratentorial healthy WM as a reference to detect the CBV and FA alterations in the lesions, although these parameters vary regionally in WM [22,42,43]. However, our measurements showed that the disease-related abnormalities can be reasonably identified and the data are consistent with previous reports. Thus, the pathology-induced changes in CBV and FA in the lesion areas are statistically evident compared with their variation in WM. Finally, we conducted a cross-sectional study. A longitudinal observation is necessary to confirm our findings and to further reveal the dynamic impact of lesion type on disease state and progression.

In summary, our data demonstrate that distinct combinations of perfusion and diffusion abnormalities characterize the status of different lesions. Substantial hemodynamic and structural impairments correspond to the T1-hypointense lesion, indicative of irreversible tissue damage, while insignificant hemodynamic and structural alterations are exhibited in the T1-isointense lesion, signifying the complex and interwoven effect resulted from the presence of coexisting pathological processes, such as tissue damage and repair. The active lesion seems more like the reactivated T1-hypointense lesion, as evidenced by its specific location and considerable tissue destruction. Lesion load represented by T1-hypointense plus active lesion volume reflects a degree of diffuse tissue injury occurring in the brain, which leads to progressive brain atrophy and accumulating irreversible disability.
